# Postoperative Heparin-Mediated Extracorporeal Low-Density Lipoprotein Fibrinogen Precipitation Aphaeresis Prevents Early Graft Occlusion after Coronary Artery Bypass Grafting

**DOI:** 10.1055/s-0036-1584167

**Published:** 2016-05-10

**Authors:** Martin Oberhoffer, Sandra Eifert, Beate Jaeger, Frithjof Blessing, A. Beiras-Fernandez, D. Seidel, B. Reichart

**Affiliations:** 1 Department of Cardiac Surgery, Asklepios Clinic St. Georg, Hamburg, Germany; 2 Department of Cardiac Surgery, Ludwig-Maximilians-University, Munich, Germany; 3 Department of Cardiothoracic Surgery, Herzzentrum Leipzig, Germany; 4 Department of Clinical Chemistry, Ludwig-Maximilians-University, Munich, Germany; 5 *Lipidzentrum Nordrhein* , Mülheim an der Ruhr, Germany; 6 Institut für Laboratoriumsmedizin, Singen, Germany; 7 Department of Cardiothoracic Surgery, JW Goethe University, Frankfurt, Germany

**Keywords:** apheresis, graft occlusion, coronary artery

## Abstract

**Background** Early graft occlusion due to thromboembolic events is a well-known complication after coronary artery bypass grafting (CABG). Fibrinogen, the coagulation factor I, is a glycoprotein that is transformed by thrombin into fibrin. It plays a major role in thrombus formation and is highly elevated after CABG. Our aim was to determine if postoperative lowering of fibrinogen levels by H.E.L.P. (heparin-mediated extracorporeal low-density lipoprotein [LDL] fibrinogen precipitation) aphaeresis could reduce the rate of early graft occlusion in patients with hypercholesterolemia undergoing CABG.

**Methods** Between December 2004 and September 2009, 36 male patients with hypercholesterolemia (mean LDL cholesterol 128 ± 12 mg/dL), mean age 58 ± 9 years, underwent CABG. Mean preoperative fibrinogen level was 387 ± 17 mg/dL. H.E.L.P. aphaeresis was postoperatively performed when fibrinogen levels exceeded 350 mg/dL on day 1 and 250 mg/dL every consecutive day up to day 8. Pre- and postaphaeresis blood samples were obtained and plasma fibrinogen level reduction was calculated. Early graft occlusion was evaluated by means of coronary angiography or multislice computed tomography before discharge.

**Results** A total of 128 distal anastomoses were performed in 36 patients (mean 3.6/patient). Postoperatively, 191 H.E.L.P. aphaeresis sessions were performed (mean 5.3/patient). Fibrinogen levels were lowered from 391 ± 10 mg/dL (preaphaeresis) to 171 ± 5 mg/dL (postaphaeresis; *p* < 0.001). Coronary angiography (multislice computed tomography in 7 patients) revealed graft patency in 125 of 128 grafts (98% patency) with three occluded venous grafts to target vessels of 1.5 mm. H.E.L.P. aphaeresis-related complications were limited to hypotensive episodes in two patients and bacteremia in one patient.

**Conclusions** H.E.L.P. apheresis offers an easy, save, and efficient method to decrease fibrinogen postoperatively in patients having CABG. Showing excellent graft patency rates in comparison to the literature, this method is a promising tool to reduce early graft occlusion after CABG.

## Background

Early graft occlusion after coronary artery bypass grafting (CABG) affects ∼15 to 25% of the grafts within 1 year after the operation, 1 2 3 and most of them become occluded within the first month. 3 4 Only 60% of vein grafts remain patent after 10 years. Early graft failure negatively influences long-term survival and freedom from myocardial infarction and redo CABG. 5 Many factors can lead to early bypass graft occlusion, but recently published work showed an increased preoperative fibrinogen level as a main predictor of both early graft occlusion and late cardiovascular events. 6 Fibrinogen, the coagulation factor I, is a glycoprotein that is transformed by thrombin into fibrin. It plays a major role in clot formation and is highly elevated after cardiac surgery as an acute phase protein. 7

Heparin-mediated extracorporeal low-density lipoprotein (LDL)/fibrinogen precipitation (H.E.L.P.) apheresis is an established method to decrease LDL cholesterol, and as a side effect fibrinogen levels are reduced by ∼50 to 70%. 8 In this prospective single-center study, we sought to determine if postoperative reduction of fibrinogen levels by H.E.L.P. aphaeresis improves early bypass graft patency after CABG.

## Materials and Methods

### Patient Characteristics and Primary Study Goals

Between December 2003 and September 2008, 36 male patients (mean age 58 ± 9 years) underwent CABG and postoperative H.E.L.P. apheresis. All patients had hypercholesterolemia (mean LDL cholesterol 128 ± 12 mg/dL) and three-vessel coronary disease. Patient demographics are given in Table 1 . The patients were on standard postoperative medication consisting of aspirin, β-blocking agents, and statins.

**Table 1:** Demographic data

Patients ( *n* )	36
Age (y), mean	58 ± 9
Male ( *n* )	36 (100%)
Coronary three-vessel-disease ( *n* )	36 (100%)
Cardiovascular risk factors ( *n* )	
Hypertension	25 (70%)
Diabetes	6 (17%)
Smoking	12 (33%)
Hypercholesterolemia	36 (100%)
Previous myocardial infarction	16 (21%)

Thirty-four patients received cardiopulmonary bypass using antegrade cardioplegia and mild hypothermia, and two patients underwent off-pump revascularization. For cardiopulmonary bypass, heparin (300 IU/kg) was administered to achieve an activated clotting time exceeding 400 seconds. Patients who were operated with the off-pump technique received 150 IU/kg, and activated clotting time was kept above 200 seconds while the anastomosis was performed. After standard median sternotomy, the thoracic internal artery and saphenous vein graft as well as radial artery (RA) grafts were harvested. The vein grafts were tested and slightly distended with Ringer's acetate, checked for leakage, and stored at room temperature. The thoracic internal and radial arteries were kept in papaverine gauze until implantation. Distal anastomoses were performed with 7–0 or 8–0 Prolene (Ethicon; Somerville, New Jersey, United States). Proximal anastomoses were done with 6–0 or 7–0 Prolene running sutures with the help of a side-biting clamp. In three veins and two radial arteries, the HEARTSTRING device (Maquet GmbH, Rastatt, Germany) was used. The graft flow was assessed intraoperatively by transit time measurement routinely. Acetylsalicylic acid was administered from postoperative day 1 on. The primary outcome measures of the study were reduction of fibrinogen and early graft patency. Several laboratory values were measured, which are not the subject of this study. Side effects and postoperative complications of the therapy were documented. All patients gave written informed consent.

### H.E.L.P. Aphaeresis Procedure

**Fig. 1:**
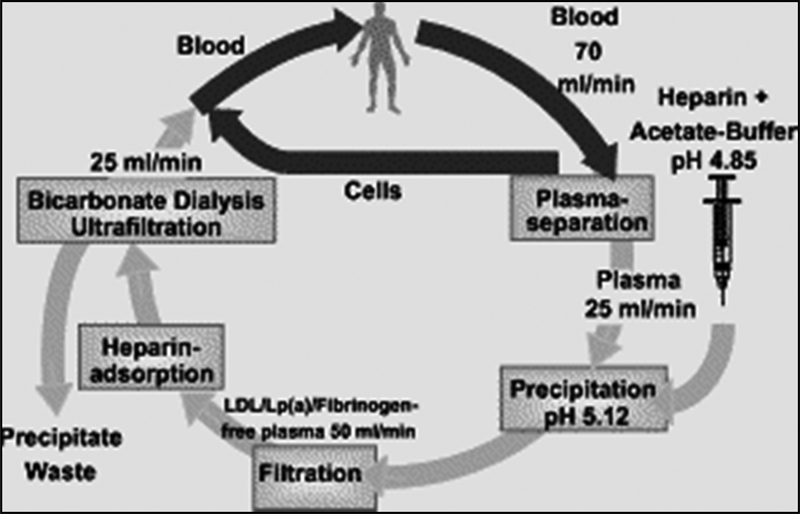
Schematic description of the heparin-mediated extracorporeal LDL fibrinogen precipitation aphaeresis procedure. Abbreviations: LDL, low-density lipoprotein; Lp(a), lipoprotein(a).

The H.E.L.P. system (Plasmat Futura, B. Braun, Melsungen, Germany) basically operates by increasing positive charges on LDL cholesterol and lipoprotein(a) (Lp[a]) molecules at a pH of 5.12 when a network of heparin and fibrinogen is formed. A detailed description of the process has been published previously. 9 Briefly, venous blood is drawn from a central venous line or a cubital vein, and plasma is obtained by a plasma separator and mixed continuously with an acetate-heparin buffer (pH 4.85). The precipitation of fibrinogen, LDL, and Lp(a) occurs immediately, and the suspension is circulated through a polyethersulfone membrane to remove the precipitate. Excess heparin is adsorbed by an anion-exchange filter, physiologic pH is restored by bicarbonate dialysis, and extra fluid is removed by ultrafiltration. Finally, plasma is mixed with the red blood cells and returned to the patient, as shown in Fig. 1 . A single H.E.L.P. session takes about 1 hour, and 3 to 3.5 L of plasma are treated.

### Blood Samples

Blood was drawn from each patient before surgery and on every postoperative day up to day 8. The fibrinogen levels were measured in citrate plasma using the method of Clauss (STA-R, Roche, Basel, Switzerland). H.E.L.P. aphaeresis was performed when the fibrinogen levels exceeded 350 mg/dL on the first postoperative day and 250 mg/dL on every consecutive day. Pre- and postaphaeresis differences were calculated.

### Statistical Analysis

All analyses were performed using the SPSS software (version 15.0 for Windows, SPSS, Inc., Chicago, Illinois, United States). Statistical descriptive analyses of the results are presented as mean values ± standard deviations. The analytical studies were performed by analysis of variance. A post hoc Tukey test for normally distributed values was used for posterior comparisons. A *p* value less than 0.05 was considered statistically significant.

## Results

### Operative Data

A total of 128 bypasses were performed in 36 patients (3.6 grafts/patients). The type and graft distribution are shown in Table 2 . The left internal thoracic artery (LITA) was anastomosed sequentially to the left anterior descending and a diagonal branch in 1 case, the RA as a T-graft proximally to a vein was anastomosed to a LITA in 1 case, the right internal thoracic artery (RITA) as a T-graft was proximally anastomosed to the LITA in 2 cases, and 1 free right internal thoracic artery was directly connected to the aorta.

**Table 2:** Perioperative data regarding coronary revascularization (bypass graft, target vessel)

Target vessel (coronary artery)	LITA	RITA	RA	Saphenous vein graft
LAD	29	0	0	4
RCX	0	2	0	12
RCA	0	4	8	17
Diagonal branch	3	2	3	19
Marginal branch	0	9	5	11
Total	32	17	16	63

Abbreviations: LAD, left anterior descending; LITA, left internal thoracic artery; RCA, right coronary artery; RCX, ramus circumflexus; RITA, right internal thoracic artery.

### H.E.L.P. Aphaeresis

**Fig. 2:**
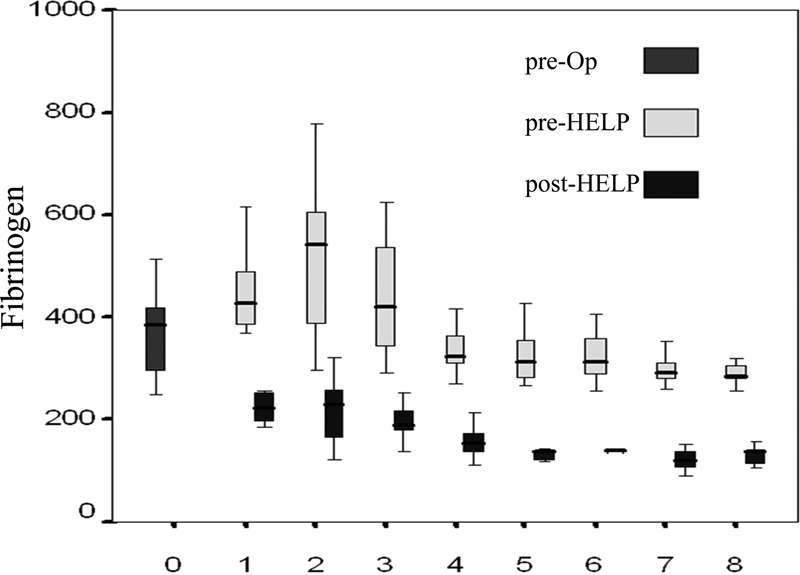
Levels of fibrinogen (mg/dL) before and after heparin-mediated extracorporeal low-density lipoprotein fibrinogen precipitation (HELP) aphaeresis.

One hundred ninety-one aphaeresis sessions were necessary (5.3/patient) in our study population. Fig. 2 demonstrates the preoperative as well as pre- and postaphaeresis fibrinogen levels. Of note, the peak on postoperative day 2 is explained by the fact that patients with a level below 350 mg/dL on postoperative day 1 (e.g., 340 mg/dL) show extremely high fibrinogen levels the following day, resulting in a mixture of very high levels of those who did not have H.E.L.P. therapy on postoperative day 1 and those who did. Fibrinogen levels were reduced from a mean of 391 ± 10 mg/dL (preaphaeresis) to a mean of 171 ± 5 mg/dL (postaphaeresis), as shown in Fig. 2 ( *p* < 0.001)—a 220 mg/dL or 52% mean reduction of fibrinogen per session.

### Bypass Graft Patency

Bypass graft patency was evaluated by conventional angiography in 29 patients and by multislice computed tomography (64-slice) in 7 patients after 11 ± 9 days. Two independent experienced investigators read the scans and judged the bypasses patent or occluded. The total bypass patency rate was 98% (125/128 grafts). All 32 LITA, 17 RITA, and 16 RA grafts were patent, and 3 vein grafts to diagonal and marginal targets of 1.5-mm diameter were occluded, reflecting a 100% patency rate for arterial grafts and a 95% patency rate for venous grafts ( Table 3 ). The occluded vein grafts were performed under cardioplegic arrest.

**Table 3:** Perioperative patency diagnostics for bypass surgery

Angiography ( *n* )	29 (81%)
MSCT ( *n* )	7 (19%)
Total	36 (100%)
Patency	
LITA ( *n* )	32/32 (100%)
RITA ( *n* )	17/17 (100%)
RA ( *n* )	16/16 (100%)
SVG ( *n* )	60/63 (95%)
Total ( *n* )	125/128 (98%)

Abbreviations: LITA, left internal thoracic artery; MSCT, multislice computed tomography; RA, radial artery; RITA, right internal thoracic artery; SVG, saphenous vein graft.

### Postoperative Complications

Postoperative complications are listed in Table 4 . The 30-day mortality was 0%. No patient suffered from new myocardial infarction or from stroke. Pericardial effusion, a possible H.E.L.P.-related heparin-mediated effect, occurred in 2 patients. One patient showed either a sternal dehiscence or pneumonia, and 4 patients had new-onset atrial fibrillation, which converted in sinus rhythm after appropriate medication. Signs of infections were highly suspicious for bacteremia due to the central venous line in 1 patient. The catheter was removed and further aphaeresis was done via a cubital vein.

**Table 4:** Postoperative outcome

Death ( *n* )	0 (0%)
Perioperative MI ( *n* )	0 (0%)
Stroke ( *n* )	0 (0%)
Pericardial effusion ( *n* )	2 (6%)
Sternal dehiscence ( *n* )	1 (3%)
Pneumonia ( *n* )	1 (3%)
Atrial fibrillation ( *n* )	4 (12%)
Bacteremia ( *n* )	1 (3%)

Abbreviation: MI, myocardial infarction.

## Discussion

Early graft occlusion is a known complication after CABG. The graft patency of bypass grafts in coronary surgery may depend on multiple factors, including age, sex, the quality of the grafts and target vessels, and even components of the coagulation cascade. 1 The thromboembolic occlusion of the bypass graft occurs at a frequency of 5 to 15%, depending on the graft utilized. 1 2 3 Fibrinogen is one component leading to early graft occlusion. As described by Blessing et al, fibrinogen is a substrate of thrombus formation that plays a major role in both primary and secondary hemostasis. 8 9 The surgical trauma itself initiates the acute-phase response and also activates the coagulation and clot formation. These factors may lead to high fibrinogen levels of up to 600 mg/dL during the early postoperative period, providing an impaired hemorheological pattern that promotes thrombus formation.

The general acceptance of fibrinogen as an important risk factor for acute cardiovascular syndromes is rather low. However, as described by Jaeger et al, 10 evidence from a recent meta-analysis formed by 22 studies of 63,736 subjects and 5,717 events suggests that the risk for myocardial infarction and stroke is almost twice as high if the fibrinogen level exceeds 3.03 g/L, with an odds ratio of 1.99 and a 95% confidence interval of 1.85 to 2.13. The predictive value of fibrinogen levels equally applies to men and women independent of age and primary or secondary prevention. As emphasized by Jaeger et al, repeated fibrinogen measurements are extremely useful specifically in high-risk patients. 10 In combination with classical cardiovascular risk factors such as hypertension, hypercholesterolemia, or diabetes, the risk of acute cardiovascular syndromes may further increase by 6- to 12-fold, and fibrinogen remains an independent risk factor for both cardiac and cardiovascular atherothrombotic complications, specifically the vulnerable plaque formation, as well as for complications after interventional or surgical treatment. Fibrinogen and its effector protein thrombin substantially determine the extent and outcome of atherothrombotic complications, because they are the molecules linking the mutually atherosclerotic events, coagulation/fibrinolysis, rheology/vasotonus, and inflammation. 9 11 In our patients, the fibrinogen levels could be postoperatively reduced by up to 50% per session. In our opinion, this reduction may be in part responsible for the graft patency rates of our coronary bypasses.

Additionally, several studies reported a cumulative effect of fibrinogen and LDL cholesterol. Interventional studies on fibrinolytic and defibrinating substances have confirmed the benefit of fibrinogen reduction and extended the experimental evidence for the relevance of fibrinogen in the pathogenesis of these syndromes. Accordingly, the preventive use of fibrates leading to moderate reductions in plasma cholesterol and fibrinogen significantly diminished the rate of reinfarction. 9 Biochemically, the composition of newly formed LDL particles after aphaeresis is described to be altered: LDL particles isolated after LDL aphaeresis had an increased resistance to oxidative stress in vitro. 12 In addition, antioxidants are not depleted by LDL aphaeresis. The extracorporeal method itself does not have a negative impact on the oxidative/antioxidative balance. A recent investigation showed that LDL cholesterol had a more pronounced effect on blood rheology than fibrinogen. 13 14

The emerging possibilities of a >50% fibrinogen reduction as noted by studies using H.E.L.P. aphaeresis strengthen the therapeutic concept of eliminating the blood risk factors—as effective as it can be—to achieve an optimal plaque regression. 15

## Conclusion

Early and extensive fibrinogen aphaeresis is a safe and very effective extracorporeal treatment to influence the outcome after CABG as shown in this study. The fibrinogen levels can be reduced by more than 60% by using aphaeresis. Additionally, LDL, Lp(a), and C-reactive protein can be reduced by this method. Furthermore, adhesion molecules and activities of inflammatory cells were also found to be reduced after a single aphaeresis session. This method demonstrates a novel therapeutic approach for preventing early graft occlusion in patients undergoing multivessel CABG.

## References

[1] Fitzgibbon G M Kafka H P Leach A J Keon W J Hooper G D Burton J R Coronary bypass graft fate and patient outcome: angiographic follow-up of 5,065 grafts related to survival and reoperation in 1,388 patients during 25 years J Am Coll Cardiol 1996 28 03 616 626 8772748 10.1016/0735-1097(96)00206-9

[2] Domanski M J Borkowf C B Campeau L Prognostic factors for atherosclerosis progression in saphenous vein grafts: the postcoronary artery bypass graft (Post-CABG) trial J Am Coll Cardiol 2000 36 06 1877 1883 11092659 10.1016/s0735-1097(00)00973-6

[3] Motwani J G Topol E J Aortocoronary saphenous vein graft disease: pathogenesis, predisposition, and prevention Circulation 1998 97 09 916 931 9521341 10.1161/01.cir.97.9.916

[4] van Brussel B L Voors A A Ernst J M Knaepen P J Plokker H W Venous coronary artery bypass surgery: a more than 20-year follow-up study Eur Heart J 2003 24 10 927 936 12714024 10.1016/s0195-668x(03)00004-6

[5] Halabi A R Alexander J H Shaw L K Relation of early saphenous vein graft failure to outcomes following coronary artery bypass surgery Am J Cardiol 2005 96 09 1254 1259 16253593 10.1016/j.amjcard.2005.06.067

[6] Hedman A Larsson P T Alam M Wallen N H Nordlander R Samad B A CRP, IL-6 and endothelin-1 levels in patients undergoing coronary artery bypass grafting. Do preoperative inflammatory parameters predict early graft occlusion and late cardiovascular events? Int J Cardiol 2007 120 01 108 114 17141340 10.1016/j.ijcard.2006.09.004

[7] Parolari A Colli S Mussoni L Coagulation and fibrinolytic markers in a two-month follow-up of coronary bypass surgery J Thorac Cardiovasc Surg 2003 125 02 336 343 12579103 10.1067/mtc.2003.2

[8] Blessing F Wang Y Walli A K Seidel D Heparin-mediated extracorporeal low-density lipoprotein precipitation: rationale for a specific adjuvant therapy in cardiovascular disease Transfus Apheresis Sci 2004 30 03 255 266 10.1016/j.transci.2004.01.009 15172631

[9] Seidel D H.E.L.P. apheresis therapy in the treatment of severe hypercholesterolemia: 10 years of clinical experience Artif Organs 1996 20 04 303 310 8860711 10.1111/j.1525-1594.1996.tb04449.x

[10] Jaeger B R Richter Y Nagel D Longitudinal cohort study on the effectiveness of lipid apheresis treatment to reduce high lipoprotein(a) levels and prevent major adverse coronary events Nat Clin Pract Cardiovasc Med 2009 6 03 229 239 19234501 10.1038/ncpcardio1456

[11] Maresca G Di Blasio A Marchioli R Di Minno G Measuring plasma fibrinogen to predict stroke and myocardial infarction: an update Arterioscler Thromb Vasc Biol 1999 19 06 1368 1377 10364066 10.1161/01.atv.19.6.1368

[12] Schettler V Wieland E [Effects of LDL-apheresis—more than reduction of cholesterol?] Dtsch Med Wochenschr 2007 132 11 575 578 17342636 10.1055/s-2007-970381

[13] Bohl S Kassner U Eckardt R Single lipoprotein apheresis session improves cardiac microvascular function in patients with elevated lipoprotein(a): detection by stress/rest perfusion magnetic resonance imaging Ther Apher Dial 2009 13 02 129 137 19379152 10.1111/j.1744-9987.2009.00667.x

[14] Stefanutti C Di Giacomo S Mazzarella B Castelli A LDL apheresis: a novel technique (LIPOCOLLECT 200) Artif Organs 2009 33 12 1103 1108 19995360 10.1111/j.1525-1594.2009.00959.x

[15] Mellwig K P van Buuren F Schmidt H K Wielepp P Burchert W Horstkotte D Improved coronary vasodilatatory capacity by H.E.L.P. apheresis: comparing initial and chronic treatment Ther Apher Dial 2006 10 06 510 517 17199883 10.1111/j.1744-9987.2006.00441.x

